# Infecciones por *Ehrlichia* spp., *Anaplasma* spp. y *Babesia* spp. en Puerto Ordaz, estado Bolívar, Venezuela

**DOI:** 10.7705/biomedica.7635

**Published:** 2025-08-11

**Authors:** Julman Rosiris Cermeño, Owen Fernando Martínez, Pedro Waykin Tong, Salvador José Penna, Thays Yraima Natera

**Affiliations:** 1 Departamento de Parasitología y Microbiología, Universidad de Oriente, Núcleo Bolívar, Ciudad Bolívar, Estado Bolívar, Venezuela Universidad de Oriente Universidad de Oriente Núcleo Bolívar Ciudad Bolívar Estado Bolívar Venezuela; 2 Departamento de Ciencias Fisiológicas, Universidad de Oriente, Núcleo Bolívar, Ciudad Bolívar, Estado Bolívar, Venezuela Universidad de Oriente Universidad de Oriente Núcleo Bolívar Ciudad Bolívar Estado Bolívar Venezuela; 3 Centro Médico Orinoco, Ciudad Bolívar, Estado Bolívar, Venezuela Centro Médico Orinoco Centro Médico Orinoco Ciudad Bolívar Estado Bolívar Venezuela; 4 Consultorio Veterinario Mi Primera Huellita Natera, Puerto Ordaz, Venezuela Consultorio Veterinario Mi Primera Huellita Natera Consultorio Veterinario Mi Primera Huellita Natera Puerto Ordaz Venezuela

**Keywords:** anaplasmosis, ehrlichiosis, babesiosis, perros, enfermedades por picaduras de garrapatas, enfermedad de Lyme., Anaplasmosis, ehrlichiosis, babesiosis, dogs, tick-borne diseases, tick-borne diseases, Lyme diseases.

## Abstract

**Introducción.:**

La prevalencia de infecciones transmitidas por garrapatas se desconoce en la mayoría de los países latinoamericanos, incluyendo a Venezuela.

**Objetivo.:**

Estimar la prevalencia de infecciones transmitidas por garrapatas en humanos y en perros en Puerto Ordaz, estado Bolívar, Venezuela.

**Materiales y métodos.:**

Se realizó un estudio exploratorio, descriptivo y prospectivo. Se evaluaron 181 individuos que aceptaron participar en forma voluntaria y dieron su consentimiento informado por escrito, y 10 perros llevados por sus dueños. Se aplicó una encuesta clínico-epidemiológica y se tomaron muestras de sangre venosa y capilar. Se hizo un frotis de capa blanca y una extensión sanguínea, que luego fueron teñidos con Giemsa.

**Resultados.:**

Se observaron infecciones transmitidas por garrapatas en el 85,1 % (n =154) de los individuos. La más frecuente fue por *Ehrlichia* spp. (n = 153; 84,5 %), seguida de *Babesia* spp. (n = 39; 21,5 %) y *Anaplasma* spp. (n = 39; 21,5 %). Las mujeres fueron las más afectadas (n = 117; 64,6 %), con un rango de edad entre los 5 y los 97 años. El 29,3 % (n = 53) de los individuos infectados presentó coinfección de *Ehrlichia* spp. y *Babesia* spp., mientras que el 6,4 % (n = 12) tuvo coinfección por los tres agentes patógenos estudiados. Todos los perros, diez en total, presentaron infecciones transmitidas por garrapatas: 10 por *Ehrlichia* spp., 5 por *Anaplasma* spp. y 5 por *Babesia* spp. Se encontró una asociación estadísticamente significativa entre la presencia de garrapatas peridomiciliares-favorecida por la falta de paseos a la mascota y la tenencia de perros enfermos o de edad avanzada-y las infecciones transmitidas por garrapatas (p < 0,05).

**Conclusiones.:**

Se evidenció una gran prevalencia de enfermedades transmitidas por garrapatas en las poblaciones humana y canina estudiadas.

## Abstract (en)

**Introduction.:**

The prevalence of tick-borne infections remains unknown in most Latin American countries, including Venezuela.

**Objective.:**

To estimate the prevalence of tick-borne infections in humans and dogs in Puerto Ordaz, Bolivar state, Venezuela.

**Materials and methods.:**

An exploratory, descriptive, and prospective study was conducted involving 181 individuals -who voluntarily agreed to participate and provided their written informed consent-and 10 dogs brought in by their owners. A clinical-epidemiological survey was conducted, and venous and capillary blood samples were collected. Buffy coat and blood smear tests were prepared and stained using Giemsa.

**Results.:**

Tick-borne infections were detected in 85.1% (n = 154) of the participants. The most frequent infection was caused by *Ehrlichia* spp. (n = 153; 84.5%), followed by Babesia spp. (n = 39; 21.5%), and *Anaplasma* spp. (n = 39; 21.5%). Women were the most affected (n = 117; 64.6%), with ages ranging from 5 to 97 years. Among infected individuals, 29.3% (n = 53) were co-infected with *Ehrlichia* spp. and Babesia spp., while 6.4% (n = 12) presented co-infection with all three pathogens under study. All the dogs (n = 10) presented tick-borne infections: 10 with *Ehrlichia* spp., 5 with *Anaplasma* spp., and 5 with *Babesia* spp. A statistically significant association was found between the presence of peridomestic ticks-favored by not walking the dog and having sick or elderly individuals-and tick-borne infections (p < 0.05).

**Conclusions.:**

A high prevalence of tick-borne diseases is evidenced, both in the studied humans and dogs.

Entre las enfermedades transmitidas por garrapatas, se destacan varias de importancia epidemiológica, como la borreliosis o enfermedad de Lyme, la ehrlichiosis, la anaplasmosis y la babesiosis. Estas son emergentes y representan un importante problema de salud pública. Mundialmente, se han adelantado numerosos estudios, sobre todo en países donde estas infecciones son de notificación obligatoria, y se ha observado que su número se ha duplicado en los últimos doce años y su distribución geográfica se ha extendido ^1^^-^^5^.

La borreliosis, o enfermedad de Lyme, es causada principalmente por el complejo *Borrelia burgdorferi* sensu lato, que comprende un conjunto de espiroquetas gramnegativas, como *B. burgdorferi, B. afzelii, B. garinii* y, más recientemente, *B. mayonii*. Estas especies tienen una amplia distribución en regiones de Europa, Norteamérica, Asia y Latinoamérica ^3^.

La ehrlichiosis incluye un grupo de enfermedades causadas por bacterias cocoides, gramnegativas e intracelulares del género *Ehrlichia*, en el cual se destacan las especies *E. chaffeensis, E. ewingii* y *E. canis*. Cada una de ellas es capaz de producir un tipo de ehrlichiosis distinta, según el huésped y el tipo de célula que parasite ^4^^,^^6^. Las infecciones transmitidas por garrapatas pueden cursar de forma asintomática o llegar a causar la muerte ^6^.

En Latinoamérica, mediante diferentes técnicas diagnósticas, se han reportado cifras variables de prevalencia de ehrlichiosis humana, como del 10,5 % en Minas Gerais al sureste de Brasil ^7^, del 3,7 % al 19,0 % en una comunidad rural en Perú ^8^, y entre 0 y 74 % en individuos sanos y con factores de riesgo en Colombia ^9^. En Venezuela, en el estado Lara, se ha documentado una prevalencia del 30 % de ehrlichiosis en personas sintomáticas y aparentemente inmunocompetentes ^10^. En el estado Aragua, se ha registrado una prevalencia del 45 % ^11^ y, en Caracas-el distrito capital-, del 13,8 % en individuos con infección por el virus de la inmunodeficiencia humana (HIV) ^12^.

La anaplasmosis es una enfermedad producida por *Anaplasma phagocytophilum* y A. *platys*, agentes patógenos intracelulares, bacterianos, gramnegativos, con una amplia distribución mundial. Estos agentes son transmitidos por vectores ixódidos mediante reservónos animales, como zorros, caballos, gatos, perros, ciervos, roedores y puercoespines ^13^. La distribución de *A. phagocytophilum* es extensa, aunque se encuentra con mayor frecuencia en África, la zona de mayor seroprevalencia (> 20 %), seguida de Asia (10-20 %), y Europa y América (< 10 %). Sin embargo, estas dos últimas regiones engloban el mayor número de personas con diagnóstico confirmado de enfermedades transmitidas por garrapatas ^14^. Por su parte, *A. platys* se ha descrito en Estados Unidos, Brasil y Venezuela, entre otros, y su infección se caracteriza por causar trombocitopenia en humanos y en animales ^15^^-^^17^.

Otra enfermedad que se transmite por la picadura de las garrapatas, pero con menor frecuencia, es la babesiosis. Esta infección puede transmitirse por transfusiones de sangre o por vía transplacentaria y es causada por parásitos protozoos intraeritrocitarios del género *Babesia*^18^. La *babesiosis* es una zoonosis de distribución mundial. *Babesia microti* es el principal agente etiológico de la babesiosis humana, endémica en el noreste y el medio oeste de Estados Unidos ^18^^,^^19^. Se han reportado otros casos en Asia, África, Australia, Europa y Suramérica ^18^^,^^20^^-^^22^. La babesiosis comparte diversas características clínicas con la malaria y puede ser fatal, particularmente, en personas mayores o inmunocomprometidas ^20^^,^^21^.

En Venezuela no se incluyen la ehrlichiosis, la anaplasmosis ni la babesiosis dentro del grupo de enfermedades de notificación obligatoria. Aunado a esto, las investigaciones sobre dichas infecciones son limitadas y, la mayoría de las veces, solo se describen casos aislados o series de casos ^12^^,^^23^^-^^27^. Por otro lado, a nivel global, la prevalencia de estas infecciones es subestimada en la mayoría de los países de Latinoamérica y, en especial, en el estado Bolívar (Venezuela), donde se desconoce.

Recientemente, en el estado Bolívar, se han descrito casos de ehrlichiosis y babesiosis ^27^. Sin embargo, no se han realizado estudios epidemiológicos relacionados con estas infecciones. Por tal razón, esta investigación tiene como objetivo determinar la prevalencia de infecciones de *Ehrlichia* spp., *Anaplasma* spp. y *Babesia* spp., en humanos y en perros de Puerto Órdaz, estado Bolívar, Venezuela.

## Materiales y métodos

Se trata de un estudio exploratorio, descriptivo, prospectivo y de corte transversal. El estudio fue realizado en Puerto Ordaz, ubicado al oeste del estado Bolívar, la capital del municipio Caroní, actualmente dividido en 11 parroquias. Caroní posee una superficie de 1.612 km^2^ y una población de 706.736 habitantes según el censo del 2014 del Instituto Nacional de Estadística (INE). El clima es tropical con lluvias abundantes, seguidas de una temporada seca, la cual es variable en algunas zonas. El rango de temperatura anual oscila entre 23,7 y 34,1 °C, y la pluviosidad, entre 820 y 1.600 mm, según el INE ^28^.

Los limites demográficos del municipio Caroní son los siguientes: al norte, el río Orinoco; al sur, el río Caroní, y los poblados de El Pao y los Rosos (pertenecientes al municipio de Piar); al este, el Delta Amacuro y el municipio Piar; y al oeste, el margen derecho del río Orinoco y el río Caroní, y el municipio de Angostura del Orinoco, antiguo municipio de Heres ^29^.

El estudio se realizó en la Primera Iglesia Evangélica Bautista de Puerto Ordaz, ubicada en el urbanismo Villa Bolivia, calle La Paz, Puerto Ordaz, estado Bolívar, Venezuela (coordenadas: 8^o^ 18’ 32,2” N; 62° 42’ 56,5” O).

Los individuos seleccionados fueron aquellos que dieron su consentimiento informado de manera voluntaria para participar en el estudio. Eventualmente, se incluyeron perros considerados como mascotas, ya que pueden contraer las enfermedades transmitidas por garrapatas y transportar estos agentes patógenos al interior de las viviendas, por lo que son un factor de riesgo de infección para las personas que conviven con ellos.

### Criterios de inclusión

Estos fueron: ser mayor de 18 años o, en caso de ser menor de edad, tener el consentimiento informado firmado por los padres; y, estar asintomático o presentar sintomatología sugestiva de enfermedad aguda. En el caso de los dueños de los perros incluidos, trasladar al animal en el momento del estudio.

### Criterios de exclusión

Se excluyeron aquellas personas con diagnóstico previo (menos de 12 meses) de enfermedades transmitidas por garrapatas y las que aportaran datos incompletos o incongruentes.

Cada uno de los participantes del estudio diligenció una encuesta en la que se recolectaron datos personales y epidemiológicos (nombres, apellidos, edad, sexo, dirección, tenencia de animales domésticos, tratamientos previos administrados a los animales), y sobre la condición socioeconómica según el método Graffar-Méndez Castellanos ^30^.

Se recopilaron los datos epidemiológicos de los perros mediante una ficha física, autorizada por el dueño, dentro del consentimiento informado. Estos datos fueron: edad, raza, sexo, estado de salud (malo: carencia de estado de salud adecuado; bueno: alimentación adecuada, sin signos de enfermedad, como diarrea, vómitos, ictericia, ataxia, pérdida de peso, fasciculaciones o cambios de comportamiento; y excelente: seguimiento veterinario periódico, alimentación y desparasitación adecuadas, y esquemas de vacunación completos), localización, datos clínicos, signos en los últimos seis meses, tratamientos previos contra garrapatas, uso de antibióticos (frecuencia y tipo), horas de permanencia dentro de la casa, y tipo de contacto, entre otros.

Previa asepsia y antisepsia, a cada uno de los sujetos se le extrajeron 5 mi de sangre venosa por punción con una aguja mariposa de calibre 23G o una jeringa de 5 ml. Estas muestras se sirvieron en tubos de ensayo estériles, al vacío, que contenían ácido etilen-diamino-tetraacético (EDTA) como anticoagulante. Las muestras se homogenizaron por inversión con el EDTA y se rotularon.

Por otra parte, se tomaron dos muestras de sangre capilar mediante una punción con una aguja estéril de calibre 23G en la yema del dedo índice. Se hizo un extendido sobre una lámina portaobjetos y se dejó secar por completo. La muestra se fijó con metanol y se rotuló. Los frotis sanguíneos fueron teñidos con Giemsa y observados al microscopio con objetivos de 40X y 100X. El examen microscópico de las muestras de sangre de humanos y animales, estuvo a cargo de dos observadores durante todo el estudio. Uno de ellos era especialista en parasitología y microbiología, con entrenamiento en enfermedades transmitidas por garrapatas en humanos y animales, y el otro, una licenciada en bioanálisis. Para verificar la presencia de *Anaplasma* spp. en plaquetas o glóbulos rojos, las láminas las examinó también un tercero, un médico veterinario.

En los perros, se rasuró y desinfectó uno de los miembros anteriores, se extrajeron de 2 a 3 mi de sangre de la vena cefálica por punción con una jeringa de 5 mi. Una vez recolectadas, las muestras se depositaron en tubos de ensayo estériles, al vacío y con EDTA. Se procedió a homogenizar y rotular una muestra a la vez.

Se hizo un frotis de capa blanca concentrada a partir de la muestra de sangre venosa de los sujetos y los perros. Se esperó que se produjera una sedimentación espontánea de los eritrocitos en el tubo (a temperatura ambiente), lo que ocurre, en general, en un lapso de minutos. La muestra sedimentada se centrifugó por 5 minutos a 2.500 rpm, para concentrar los leucocitos y las plaquetas. Se elaboraron los frotis sanguíneos con una fracción de la capa blanca, se dejaron secar sobre láminas portaobjetos (pulidas y libres de grasa), se fijaron con metanol y se rotularon. Los frotis de capa blanca se tiñeron con Giemsa y se examinaron con ayuda de un microscopio óptico y objetivos de 40X y 100X ^24^^,^^25^.

En los frotis de capa blanca y sangre capilar, se buscaron mórulas intracitoplasmáticas de *Ehrlichia* spp., con tropismo por monocitos o linfocitos (posiblemente *E. canis* o *E. chaffeensis*) y por granulocitos (posiblemente *E. ewingii* u otras especies); o de mórulas de *Anaplasma* spp., con tropismo por plaquetas (posiblemente *A. platys*) o glóbulos rojos (posiblemente *A. bovis* u otras especies). Para la identificación de *Babesia* spp., se buscaron trofozoítos individuales de *Babesia* spp. o dispuestos en estructuras en forma de cruz dentro de los glóbulos rojos.

Para cuantificar los valores hematológicos, se emplearon aproximadamente 20 pl de sangre total con anticoagulante. Este volumen fue procesado en el equipo analizador hematológico Mindray BC-5380™ (Mindray Shenzhen Mindray BioMedical Electronics Co., Ltd).

### Análisis estadístico

Los resultados se analizaron con el paquete estadístico *Statistical Package for the Social Sciences™* (SPSS), versión 21.0, para Windows. Las variables cualitativas se expresaron mediante frecuencias absolutas y la proporción de cada una de las categorías; las variables cuantitativas se representaron con medias y desviaciones estándar. La prueba de ji al cuadrado (χ2) y la exacta de Fisher se utilizaron para comparar las variables cualitativas. El nivel de significancia utilizado fue de p ≤ 0,05. Para las variables cuantitativas, se empleó la prueba t de Student. Se calculó la razón de probabilidades (*odds ratio*, OR) con el intervalo de confianza (IC) del 95 % para evaluar la asociación entre variables nominales dicotómicas. Se consideró un factor de riesgo o exposición, un OR mayor de 1,0.

### Aspectos éticos

Este estudio fue aprobado por la Comisión de Tesis de Grado de la Universidad de Oriente, Núcleo Bolívar, que usualmente revisa aspectos éticos y metodológicos de trabajos de investigación (Trabajo de Grado Medicina, Universidad de Oriente -TGM-2022-12-20,13/10/2022).

A las personas infectadas y sintomáticas, se les indicó un tratamiento específico para cada agente infeccioso y un control posterior. A las personas asintomáticas se les indicó un hemograma completo, pruebas de función renal y hepática, y consulta con medicina interna, medicina tropical o infectología para definir el abordaje terapéutico.

A los animales infectados, también se les prescribió tratamiento específico. Además, se realizaron charlas de prevención de infecciones transmitidas por garrapatas a humanos y animales.

## Resultados

Se evaluaron 181 individuos que aceptaron participar de forma voluntaria en el estudio. En el cuadro 1 se muestran las características epidemiológicas de la población estudiada. La edad de los participantes osciló entre los 5 y los 97 años, con una media de 46,8 ± 6,21 años. El grupo etario más frecuente (n = 45; 24,9 %) fue el de 51 a 60 años. La mayoría (n = 117; 64,6 %) de los individuos fueron mujeres. El 91,2 % (n = 165) de los participantes residía en Puerto Ordaz en el municipio de Caroní y el 8,8 % (n = 16) restante vivía en Ciudad Bolívar en el municipio de Angostura del Orinoco. La mayoría (n = 124; 68,5 %) de los sujetos de Puerto Ordaz procedían de la parroquia Cachamay, mientras que los de Ciudad Bolívar (n = 8; 4,4 %) procedían de las parroquias Agua Salada y Vista Hermosa. La ocupación más frecuente (n = 35; 19,3 %) fue la de estudiante, seguida de ama de casa (n = 26; 14,4 %) y comerciante (n = 21; 11,6 %). El grado de instrucción predominante fue el universitario (n = 62; 34,4 %), seguido de la educación secundaria (n = 42; 23,2 %), la primaria (n = 35; 19,3 %), la técnica superior (n = 19; 10,5 %), la de licenciado (n = 12; 6,6 %) y la preescolar (n = 11; 6,1 %).

**Cuadro 1:** Características epidemiológicas de los individuos estudiados según el agente etiológico transmitido por garrapatas, Puerto Ordaz, municipio Caroní, estado Bolívar

Características epidemiológicas		Infección por							
		n (%)		***Ehrlichia* spp.**		***Anaplasma* spp.**		**B*abesia* spp.**	
				n	(%)	n	(%)	n	(%)
Grupos de edad (años) 0-10		7	(3,9)	6	(3,9)	3	(7,7)	2	(3,7)
	11-20	17	(9,4)	12	(7,8)	2	(5,1)	7	(13,0)
	21-30	24	(13,3)	24	(15,7)	5	(12,8)	11	(20,4)
	31-40	7	(3,9)	7	(4,6)	3	(7,7)	2	(3,7)
	41-50	35	(19,3)	28	(18,3)	8	(20,5)	11	(20,4)
	51-60	45	(24,9)	39	(25,5)	5	(12,8)	12	(22,2)
	61-70	31	(17,1)	24	(15,7)	7	(17,9)	5	(9,3)
	71-80	13	(7,2)	11	(7,2)	6	(15,4)	3	(5,6)
	81-90	1	(0,6)	1	(0,7)	0	(0)	0	(0)
	>90	1	(0,6)	1	(0,7)	0	(0)	1	(1,9)
	Total	181	(100)	153	(100)	39	(100)	54	(100)
Sexo									
	Masculino	64	(35,4)	53	(34,6)	12	(30,8)	20	(37,0)
	Femenino	117	(64,6)	100	(65,4)	27	(69,2)	34	(63,0)
	Total	181	(100)	153	(100)	39	(100)	54	(100)
Procedencia (parroquia) Municipio Caroní									
Sector Puerto Ordaz									
	Cachamay	124	(68,5)	100	(65,3)	31	(79,4)	32	(59,2)
	Unare	16	(8,8)	16	(10,5)	4	(10,3)	3	(5,5)
	Universidad	16	(8,8)	13	(8,5)	4	(10,3)	6	(11.1)
Sector San Félix									
	11 de Abril	5	(2,8)	4	(2,6)	0	(0)	1	(1,8)
	Simón Bolívar	2	(1.1)	2	(1.3)	0	(0)	0	(0)
	Vista al Sol	1	(0,6)	1	(0,6)	0	(0)	0	(0)
	Dalla Costa	1	(0,6)	0	(0)	0	(0)	0	(0)
	Total	165	(91,2)	136	(88,8)	39	(100)	42	(77,6)
Municipio Angostura del Orinoco									
	Agua Salada	8	(4,4)	8	(5,2)	0	(0)	4	(7,4)
	Vista Hermosa	8	(4,4)	8	(5,2)	0	(0)	8	(14,8)
	Total	16	(8,8)	16	(10,4)	0	(0)	12	(22,2)
Ocupación									
	Estudiante	35	(19,3)	29	(18,9)	9	(23,0)	15	(27,8)
	Ama de casa	26	(14,4)	21	(13,7)	6	(15,3)	5	(9,3)
	Comerciante	21	(11,6)	19	(12,4)	6	(15,3)	7	(12,9)
	Jubilado	14	(7,7)	13	(8,5)	2	(5,1)	3	(5,5)
	Administrador	10	(5,5)	8	(5,2)	2	(5,1)	3	(5,5)
	Obrero	9	(5)	6	(3,9)	0	(0)	1	(1,9)
	Desempleado	9	(5)	7	(4,6)	2	(5,1)	6	(11.1)
	Médico	7	(3,9)	6	(3,9)	1	(2,5)	3	(5,5)
	Docente	6	(3,3)	4	(2,6)	1	(2,5)	2	(3,7)
	Otros	44	(24,3)	40	(26,1)	10	(25,6)	9	(16,6)
	Total	181	(100)	153	(84,5)	39	(21,5)	54	(29,8)

Otros: agricultor, supervisor, abogado, periodista, enfermero, ingeniero, contador, diseñador gráfico, estilista, bioanalista, cocinero, herrero, carpintero, técnico informático, operador, modista

Las comorbilidades registradas fueron: hipertensión arterial (n = 43; 23,76 %), diabetes mellitus (n = 10; 5,53 %), enfermedades tiroideas como hipertiroidismo e hipotiroidismo (n = 7; 3,86), enfermedad pulmonar obstructiva crónica (n = 6; 3,31 %), artrosis (n = 4; 2,20 %), artritis (n = 4; 2,20 %) y otras, como glaucoma, dermatitis seborreica, toxoplasmosis, vitiligo, pénfigo vulgar, enfermedad de Parkinson, lupus eritematoso sistémico, arritmia supraventricular, osteoporosis, insuficiencia venosa y fibromialgia (n = 12; 6,62 %). Solo un paciente asintomático refirió haber tenido ehrlichiosis (n = 1; 0,55 %) hacía más de dos años. El resto de los sujetos (n = 118; 65,2 %) no refirió antecedentes médicos de importancia.

Durante el estudio, la mayoría de los sujetos eran asintomáticos (n = 175; 96,7 %); los sintomáticos (n = 6; 3,3 %) refirieron manifestaciones clínicas inespecíficas en los últimos seis meses, como pérdida de peso, alteración de la memoria, dolor articular, cefalea, alteración del estado de ánimo, fatiga, sofocos, fiebre, mareos, debilidad general y astenia.

Las infecciones transmitidas por garrapatas, causadas, al menos, por un agente patógeno, se detectaron en el 85,1 % (n = 154) de los individuos estudiados. La infección más frecuente fue por *Ehrlichia* spp. (n = 153; 84,5 %), seguida por *Babesia* spp. (n = 54; 29,8 %) y *Anaplasma* spp. (n = 39; 21,5 %).

En el cuadro 1 se muestran las infecciones transmitidas por garrapatas según grupo etario, sexo, procedencia y ocupación de la población evaluada. El grupo de 51 a 60 años presentó mayor frecuencia de infecciones por *Ehrlichia* spp. (n = 39; 25,4 %) y *Babesia* spp. (n = 12; 22,2 %). El menor porcentaje de infecciones (n = 2; 1,4 %; p > 0,05), se presentó en el grupo de mayores de 81 años. La mayoría de las infecciones por *Anaplasma* spp. (n = 8; 20,5 %) se presentó en el grupo de 41 a 50 años y fueron menos frecuentes en aquellos mayores de 81 años (p > 0,05). Las infecciones por Ehrlichia spp. (n = 100; 65,4 %), *Anaplasma* spp. (n = 27; 69,2 %) y *Babesia* spp. (n = 34; 62,9 %) fueron predominantes en las mujeres (p > 0,05).

**Figura 1.**
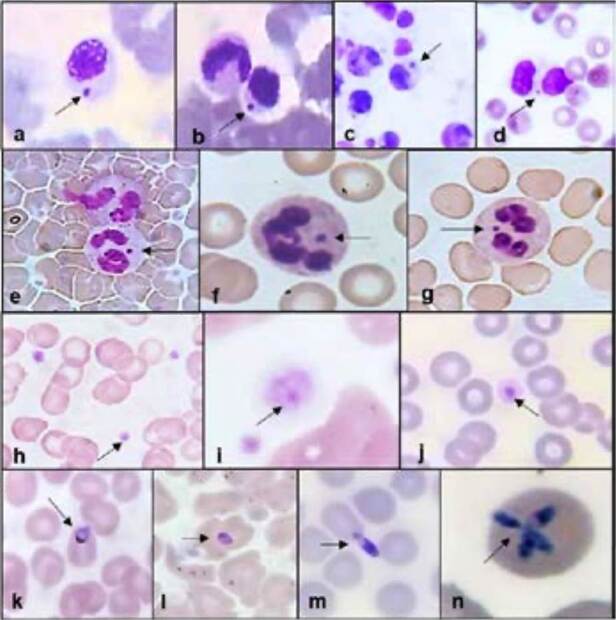
a, b, c y d: mórulas posiblemente de *Ehrlichia* canis o *E. chaffeensis* en leucocitos mononucleares; e, f y g: mórulas intracelulares posiblemente de *E. ewingii* en leucocitos polimorfonucleares; h, i y j: mórulas intraplaquetarias posiblemente de *Anaplasma* platys; k, l y m: trofozoítos de *Babesia* spp; n: estructura en forma de cruz por *Babesia* spp. observada dentro de los glóbulos rojos mediante tinción de Giemsa y visualizada a un aumento de 1.000X.

En la parroquia Cachamay se detectaron infecciones trasmitidas por garrapatas causadas por los tres agentes patógenos evaluados en este estudio. La infección más frecuente fue por *Ehrlichia* spp (n = 100; 65,3 %), seguida de *Babesia* spp. (n = 32; 59,2 %) y *Anaplasma* spp. (n = 31; 79,4 %). La mayoría de los individuos fueron asintomáticos (n = 175; 96,7 %) y, de ellos, 150 tenían infección por *Ehrlichia* spp. (82,8 %), 38 por *Anaplasma* spp. (20,9 %) y 52 por *Babesia* spp. (28,7 %) (figura 1). Entre los individuos con síntomas inespecíficos (n = 6; 3,3 %), tres presentaron infección por *Ehrlichia* spp. (*X* ^2^ = 5,659; gl = 1; p = 0,001); dos por *Babesia* spp. y uno por *Anaplasma* spp. (p > 0,05).

Respecto a las coinfecciones, el 29,3 % (n = 53) de los individuos presentó coinfección por *Ehrlichia* spp. y *Babesia* spp., mientras que solo el 6,4 % (n = 12) presentó coinfección por los tres microorganismos. Asimismo, no hubo asociación entre el grado de instrucción y las infecciones evaluadas.

En los sujetos evaluados, el valor promedio de hemoglobina fue de 13,13 ± 1,36 g/dl y el de hematocrito fue de 41,33 % ± 4,20; el 9,4 % (n = 17) de los participantes tenía anemia. El promedio de leucocitos de la población incluida fue de 10.651 ± 53.061 células/ml; el 23,2 % (n = 42) de los individuos tenía leucopenia y, el 1,1 % (n = 2), leucocitosis. El promedio de plaquetas fue de 247.697 ± 85.624 células/ml y el 5 % (n = 9) de la población estudiada presentó trombocitopenia. El 61,33 % de los individuos no presentó alteraciones en los valores hematológicos (n = 111). No se encontró una relación estadísticamente significativa entre los valores hematológicos y las infecciones transmitidas por garrapatas (p > 0,05).

Respecto a los factores de exposición en humanos (cuadro 2), se evidenció que el 75,1 % (n = 136) de los individuos estudiados visitaba frecuentemente casas donde había perros, el 69,6 % (n = 126) de los sujetos había tenido contacto cercano con perros en cualquier momento de su vida, y el 58 % (n = 105) vivía con perros en su domicilio. Se identificó una asociación entre la visita a viviendas con perros y las infecciones por *Ehrlichia* spp. (*X* ^2^ = 3,549; gl = 1), aunque no fue estadísticamente significativa (p = 0,060).

**Cuadro 2.** Relación entre los factores de exposición y las infecciones transmitidas por garrapatas en humanos, Puerto Ordaz, municipio Caroní, estado Bolívar

Factores de exposición	n (%)	*Ehrlichiosis*		OR*	p**	*Babesiosis*		OR*	P	*Anaplasmosis*		OR*	p
		Pos n (%)	Neg n (%)	(IC _95%_)		Pos n (%)	Neg n (%)	(IC _95%_)		Pos n (%)	Neg n (%)	(IC _95%_)	
Perros en casa***	105 (58)	88 (48,6)	17 (9,4)	0,87	0,75	34 (18,8)	71 (39,2)	0,38	1,34	24 (13,3)	81 (44,8)	1,20	0,61
				(0,38-1,99)					(0,69-2,58)			(0,58-2,49)	
Contacto cercano con perros****	126 (69,6)	108 (59,7)	18(9,9)	1,33	0,51	38 (21)	88 (48,6)	0,89	1,05	26 (14,4)	100 (55,2)	0,84	0,65
				(0,57-3,11)					(0,53-2,10)			(0,39-1,79)	
Visita a casas con perros	136 (75,1)	111 (61,3)	25 (13,8)	0,32	0,06	42 (23,2)	94 (51,9)	0,59	1,2	26 (14,4)	110(60,8)	0,58	0,17
				(0,09-1,11)					(0,58-2,61)			(0,27-1,26)	
Picadura de garrapatas (en cualquier momento de la vida)	50 (27,6)	41 (22,7)	9(5)	0,77	0,56	16(8,8)	34(18,8)	0,69	1,15	9(5)	41 (22,7)	0,74	0,47
				(0,32-1,85)					(0,57-2,33)			(0,32-1,69)	
Visita a zonas rurales	37 (20,4)	31 (17,1)	6 (3,3)	0,93	0,89	12(6,6)	25(13,8)	0,70	1,17	7 (3,9)	30 (16,6)	0,82	0,66
				(0,35-2,50)					(0,54-2,53)			(0,33-2,03)	
Realización de actividades al aire libre regularmente	82 (45,3)	67 (37)	15(8,3)	0,68	0,34	20 (11)	62 (34,4)	0,15	0,62	18(9,9)	64 (35,4)	1,05	0,90
				(0,30-1,52)					(0,32-1,19)			(0,51-2,13)	
Presencia de garrapatas en casa	71 (39,2)	56 (30,9)	15(8,3)	0,50	0,09	20 (11)	51 (28,2)	0,69	0,88	17(9,4)	54 (29,8)	1,26	0,53
				(0,22-1,13)					(0,46-1,69)			(0,61-2,58)	
Presencia de Dentro de la casa	26 (14,4)	15 (8,3)	11 (6,1)			5 (2,8)	21 (11,6)			7 (3,9)	19(10,5)		
garrapatas Fuera de la casa	32 (17,7)	29 (16)	3(1,7)		0,001	9(5)	23 (12,7)	0,368		7 (3,9)	25(13,8)		0,892
Dentro y fuera de la casa	13 (7,2)	12(6,6)	1 (0,6)			6 (3,3)	7 (3,9)			3(1,7)	10 (5,5)		

Pos: positivo; Neg: negativo; OR: *odds ratio*

* Se consideró un valor mayor de 1 como factor de riesgo

** Valores inferiores se consideraron como protectores.

Se consideró significativo un valor de p <0,05.

*** Presencia del animal en la misma casa ya sea adentro o afuera

**** Personas que tienen afición, atracción u obsesión hacia el animal y duermen, besan o abrazan a sus mascotas

El 39,2 % (n = 71) de los individuos reportó la presencia de garrapatas en su domicilio: el 17,7 % (n = 32) las observó en el peridomicilio y, el 14,4 % (n = 26), en el intradomicilio. Se encontró una asociación estadísticamente significativa entre la presencia de garrapatas en el exterior de la vivienda y las infecciones por *Ehrlichia* spp. en humanos (*X* ^2^ = 16,953; gl = 3; p = 0,001).

El nivel socioeconómico más frecuente fue el estrato II (42 %; n = 76), clasificado como la clase media-alta, seguido por el estrato III (24,3 %; n = 44), el estrato I (18,2 %; n = 33) y el estrato IV (15,5 %; n = 28). Aunque los estratos II y III presentaron la mayor proporción de infecciones transmitidas por garrapatas [33,14 % (n = 60) y 22,65 % (n = 41)], no hubo una asociación estadísticamente significativa entre el nivel socioeconómico y la presencia de infecciones en los sujetos evaluados (p > 0,05).

La mayoría (n = 105) de los participantes refirió tener, al menos, un perro en su vivienda. La edad de las mascotas osciló entre los 0 y los 15 años, con una media de 5,95 ± 3,36 años. El grupo etario canino más frecuente fue el de 3 a 5 años (n = 46; 44,2 %). La mayoría de los perros eran machos (n = 40; 22,1 %), de raza mestiza (n = 44; 41,9 %) y se encontraban en buen estado de salud-según sus dueños-en el momento de la entrevista (n = 72; 68,8 %). Se encontró una asociación entre la tenencia de perros entre los 3 y los 5 años, y las infecciones causadas por *Babesia* spp. en humanos (*X* ^2^ = 9,930; gl = 3; p = 0,019). Las características epidemiológicas y clínicas de los perros se muestran en el cuadro 3.

**Cuadro 3.** Características epidemiológicas y estado de salud de los perros estudiados en Puerto Ordaz, municipio Caroní, estado Bolívar

Características epidemiológicas		n	(%)
Grupos de edad (años)			
	0-2	23	(21,2)
	3-5	46	(44,2)
	6-8	23	(22,1)
	9-12	5	(4,8)
	> 13	8	(7,7)
	Total	105	(100)
Sexo			
	Macho	40	(61,9)
	Hembra	65	(38,1)
	Total	105	(100)
Raza			
	Mestizo	44	(41,9)
	Poodle	25	(23,8)
	Schnauzer	9	(8,6)
	Yorkshire Terrier	6	(5,7)
	Pinscher miniatura	5	(4,8)
	Otras*	16	(15,2)
Tipo de alimentación			
	Alimento comercial o concentrado	10	(9,5)
	Comida de mesa	53	(50,5)
	Mixto	42	(40)
Estado de salud			
	Malo	7	(6,7)
	Bueno	72	(68,6)
	Excelente	26	(24,8)
Lugar donde duerme Dentro de la casa			
	Sala	22	(21)
	Cama con el dueño	16	(15,2)
	Pasillo	7	(6,7)
	Cuarto del dueño	5	(4,8)
Fuera de la casa			
	Patio	33	(31,4)
	Porche	22	(21)
	Total	105	(100)

* Otras: chihuahua, golden retriever, jack Russell terrier, labrador, pastor alemán, pequinés, pastor persa, pitbull terrier, pug, rottweiler

De los 105 perros que vivían con sus dueños, solo 10 fueron llevados para participar en el estudio. Se encontró que las 10 mascotas presentaban infecciones, de las cuales 5 fueron por *Ehrlichia* spp., y las otras 5 por *Anaplasma* spp. y *Babesia* spp.

Según la información proporcionada durante la entrevista, relacionada con las infecciones transmitidas por garrapatas en los perros, el 53,3 % de los dueños indicó que habitualmente sus mascotas no tenían garrapatas en el cuerpo (n = 56). En aquellos perros que solían tener garrapatas, estas eran tanto de cuerpo blando como de cuerpo duro (n = 37; 75,5 %) (cuadro 4). Se encontró una relación estadísticamente significativa entre las infecciones por *Babesia* spp. en humanos y no pasear al perro (*X* ^2^ = 6,585; gl = 1; p = 0,010). Se identificó que la presencia de signos de enfermedad en los perros, como convulsiones, diarrea, pérdida de peso y vómitos, se encuentra relacionada con las infecciones por *Ehrlichia* spp. (*X* ^2^ = 8,000; gl = 3; p < 0,046).

**Cuadro 4.** Variables relacionadas con las infecciones transmitidas por garrapatas en los perros estudiados en Puerto Ordaz, municipio Caroní, estado Bolívar

Variables		n	(%)
Número de garrapatas en el cuerpo			
	Sin garrapatas	56	(53,3)
	1-5	20	(19)
	6-10	14	(13,3)
	11-15	9	(8,6)
	16-20	5	(4,7)
	21-25	1	(1)
	Total	105	(100)
Tipo de garrapata			
	Cuerpo blando	10	(20,4)
	Cuerpo duro	2	(4,1)
	Cuerpo blando y duro	37	(75,5)
	Total	49	(100)
Aplicación de garrapaticidas en perro			
	Sí	50	(47,6)
	No	55	(52,4)
	Total	105	(100)
Tipo de garrapaticida			
	Ivermectina	22	(44)
	Fluralaner (Bravecto)	18	(36)
	Amitraz	6	(12)
	Otros*	4	(8)
	Total	50	(100)
Uso de antibióticos en los últimos seis meses			
	Sí**	7	(6,7)
	No	98	(93,3)
	Total	105	(100)
Salida de paseo con sus dueños			
	Sí	59	(56,2)
	No	46	(43,8)
	Total	105	(100)
Control veterinario en los últimos seis meses			
	Sí	26	(24,8)
	No	79	(75,2)
	Total	105	(100)
Signos clínicos en los últimos seis meses			
	No	97	(92,3)
	Sí		
	Vómitos	5	(4,76)
	Diarrea	1	(0,9)
	Convulsiones	1	(0,9)
	Pérdida de peso	1	(0,9)
	Total	105	(100)

* Fipronil, collar garrapaticida

** El antibiótico usado en los siete casos fue doxiciclina.

No se observaron diferencias estadísticamente significativas entre las personas con perros en su vivienda y el riesgo de infecciones por *Anaplasma* spp. (OR = 1,20; IC_95%_: 0,58 a 2,49) o *Babesia* spp. (OR = 1,34; 1C 95%: 0,69 a 2,58); tampoco se identificó un mayor riesgo de infección por *Ehrlichia* spp. (OR = 1,83; IC_95%_: 0,62 a 5,39) o *Anaplasma* spp. (OR = 1,13; IC_95%_: 0,45 a 2,81) en los individuos que no administraban garrapaticidas a sus mascotas. Asimismo, quienes aplicaban garrapaticidas en sus casas no tuvieron un mayor riesgo de infección por *Ehrlichia* spp. (OR = 1,39; IC_95%_: 0,47 a 4,10) ni *Babesia* spp. (OR = 1,63; IC_95%_: 0,71 a 3,72).

El no administrar antibióticos a los perros en los últimos seis meses se identificó como un factor de riesgo para infecciones en sus dueños por *Ehrlichia* spp. (OR = 1,21; IC_95%_: 1,11 al ,33), *Babesia* spp. (OR = 5,95; IC _95%_: 1,09 a 31,44) y *Anaplasma* spp. (OR = 1,38; 1C _95%_: 0,25 a 7,62) en sus dueños. No se encontró ninguna asociación entre las demás variables relacionadas con infecciones transmitidas por garrapatas en perros y las infecciones en humanos.

Los individuos cuyas mascotas fueron evaluadas estaban infectados por *Ehrlichia* spp. (n = 10), *Babesia* spp. (n = 4) y *Anapiasma* spp (n = 3). No se evidenció un mayor riesgo de infecciones por *Anapiasma* spp., en los participantes que convivían con perros infectados con *Babesia* spp. (OR = 2,67; IC_95%_: 0,16 a 45,14) o *Anapiasma* spp. (OR = 2,66; IC_95%_: 0,16 a 45,14) (cuadro 5). No se encontró una asociación estadísticamente significativa entre las demás infecciones detectadas en los perros y las infecciones en sus dueños.

**Cuadro 5.** Infecciones en individuos evaluados y sus perros en Puerto Ordaz, municipio Caroní, estado Bolívar

Infecciones en perros	Infecciones en humanos									
	n (%)	*Ehrlichiosis*		OR*	*Babesiosis*		OR	*Anapiasmosis*		OR
		Positivo n (%)	Negativo n (%)	(IC_95%_)	Positivo n (%)	Negativo n (%)	**(IC**_95%_ **)**	Positivo n (%)	Negativo n (%)	(IC_95%_)
*Babesiosis*	5(50)	5(50)	0(0)	1 **	2(20)	3(30)	1**	2(20)	3(30)	2,67 (0,16-45,14)
*Ehrlichiosis*	10(100)	10(100)	0(0)	1 **	4(40)	6(60)	1**	3(30)	7(70)	1 **
*Anapiasmosis*	5(50)	5(50)	0(0)	1 **	2(20)	3(30)	1**	2(20)	3(30)	2,66 (0,16-45,14)

OR: *odds ratio*

* Se consideró un valor mayor de 1 como factor de riesgo o exposición

** Un valor igual a 1 se consideró nulo.

## Discusión

En pocos estudios se ha investigado la prevalencia de infecciones transmitidas por garrapatas en humanos. En Venezuela, por ejemplo, solo existen publicaciones de casos aislados ^10^^,^^12^^,^^23^^-^^25^^,^^27^^,^^31^^-^^33^. En el presente estudio se determinó una prevalencia elevada (85 %) de enfermedades transmitidas por garrapatas. *Ehrlichia* spp. fue el agente etiológico más frecuente, seguido de *Babesia* spp. (30 %) y *Anapiasma* spp. (21 %). A diferencia de estos resultados, en Cuba se ha reportado una baja prevalencia de infecciones por *Anapiasma* spp. (7,2 %), *Ehrlichia* spp. (3,6 %) y *Babesia* spp. (11,5 %), mediante una metodología diferente ^34^.

La alta prevalencia de infecciones transmitidas por garrapatas se podría explicar por el contacto cercano de la población evaluada con perros, las visitas frecuentes a zonas campestres y los antecedentes de picaduras de garrapatas, todos ellos factores de riesgo para contraer este tipo de infecciones ^9^^,^^11^^,^^13^^,^^14^^,^^18^^-^^22^. Otra posibilidad es el incremento de las poblaciones de garrapatas en las áreas urbanas y el clima tropical de la zona estudiada, con altas temperaturas la mayor parte del año. Estas condiciones podrían favorecer la actividad de las garrapatas, lo que intensificaría el acecho a hospederos para su alimentación e, incluso, aumentaría su afinidad por los huéspedes humanos ^35^.

En el presente estudio, se evidenció la coinfección de múltiples agentes patógenos en perros y humanos. La más prevalente fue la de *Ehrlichia* spp. y *Babesia* spp., seguida por la coinfección triple *Ehrlichia* spp., *Anapiasma* spp. y *Babesia* spp.. Es probable que esta última sea frecuente debido a que los tres agentes patógenos son transmitidos por el mismo vector. Estas coinfecciones también se han reportado en otros estudios ^36^^-^^38^. En Colombia, se reportó la coinfección por *Babesia* spp. y *Ehrlichia* spp., en dos pacientes con síntomas clínicos ^38^. Asimismo, en Brasil, se ha documentado la coinfección por *B. burgdorferi* y *B. bovis*^39^. En Europa, Asia y Norteamérica, la coinfección de *Anapiasma* spp., *Borrelia* spp. y *Babesia* spp., se ha descrito como la más prevalente ^36^.

Las comorbilidades reportadas en este estudio -excepto las afecciones tiroideas y la artrosis- coinciden con las enfermedades crónicas señaladas en otras investigaciones similares ^40^^-^^43^. Considerando que la mayoría de los individuos eran asintomáticos y no habían sido diagnosticados con ehrlichiosis, anaplasmosis o babesiosis, estas comorbilidades podrían ser un factor de riesgo para el desarrollo de formas graves de la infección en condiciones de inmunodepresión ^44^.

Se encontró una asociación entre la presencia de garrapatas fuera de la vivienda y las infecciones por *Ehrlichia* spp. (p < 0,001) en humanos. Este hallazgo podría estar relacionado con la ecología de *Riphicephalus sanguineus*^45^^,^^46^, un artrópodo peridomiciliario debido a las condiciones propicias de humedad y temperatura del área.

Bouchard *et al*. describieron un aumento de la densidad poblacional de garrapatas debido a su capacidad de alcanzar mayores latitudes. Estos cambios de temperatura y pluviosidad inducen comportamientos alimentarios más agresivos, que se traducen en mayor actividad hematófaga, mayor frecuencia de alimentación y mayor acecho o búsqueda de huéspedes. El cambio climático no solo afecta a los vectores, sino que, también, favorece la reproducción de los reservorios y, por ende, aumenta el número de huéspedes disponibles para la alimentación de las garrapatas ^35^.

La densidad poblacional de las garrapatas en determinadas zonas geográficas podría estar asociada con los patrones migratorios de aves que transportan estos vectores. Según variables como cambios de temperatura, disponibilidad de recursos alimenticios y otros factores endógenos, las aves se desplazan grandes distancias y en el proceso liberan garrapatas previamente adheridas a ellas, lo que aumenta la probabilidad de infección en huéspedes vertebrados ^47^.

El sexo masculino, las edades extremas, los climas cálidos, la ubicación geográfica, el nivel socioeconómico y cultural, la exposición frecuente a entornos silvestres, el aumento en el número de reservorios y la domiciliación de animales han sido reportados por otros autores como factores de riesgo para las infecciones transmitidas por garrapatas ^44^^,^^48^, lo cual coincide con algunos de los factores descritos en la población evaluada.

La mayor proporción de mujeres infectadas en este estudio, podría estar relacionada con el predominio de participantes del sexo femenino. Esto contrasta con lo publicado en otros estudios ^48^, que indican mayor propensión de los hombres a las infecciones transmitidas por garrapatas, ya que, por sus ocupaciones, se encuentran más expuestos a zonas de gran riesgo, como entornos rurales con infestación de garrapatas ^27^^,^^48^.

Respecto a los grupos etarios, las infecciones predominaron en personas mayores de 60 años, posiblemente por mayor exposición a múltiples factores de riesgo a lo largo de sus vidas, similar a lo descrito por otros autores ^49^^,^^50^.

Los factores de riesgo para las enfermedades transmitidas por garrapatas han sido ampliamente descritos en la población canina, a diferencia de lo sucedido en la población humana ^14^^,^^44^^,^^48^. Algunos estudios afirman que, en los humanos, variables como el sexo, la edad y la raza, no Influyen en el riesgo de infecciones transmitidas por garrapatas. Por el contrario, la edad del perro sí es un factor determinante ^51^^-^^53^. Según algunos Investigadores, el envejecimiento en la población canina se inicia a partir de los 7 años y se consideran perros geriátricos a aquellos mayores de 11 años. A esta edad, se estima que el perro ha tenido una exposición acumulada a infestaciones por garrapatas, además de presentar un deterioro progresivo del sistema inmunológico, propio de la edad ^53^^,^^54^.

La mayoría de los perros eran adultos y solo el 30 % eran geriátricos. Todos resultaron infectados, al menos, con un agente patógeno transmitido por garrapatas. Se estima que los dueños de perros mayores de seis años tienen mayor riesgo de infección por *Babesia* spp. ^51^^-^^53^.

Por otro lado, la cantidad de perros en el domicilio se considera como un factor de riesgo ^53^. La tenencia de más de una mascota en el hogar predispone a los animales a infecciones por *Anaplasma* spp., ya que la densidad poblacional canina es directamente proporcional a la prevalencia e infestación de *R. sanguineus*, vector transmisor de *A. platys*. Esto se debe a que una mayor cantidad de huéspedes aumenta la probabilidad de alimentación y reproducción rápida de las garrapatas. Asimismo, el estilo de vida del perro es un factor de riesgo. Los perros callejeros tienen mayor riesgo de ser infectados por *E. canis*, en comparación con los domesticados que viven en un domicilio ^52^. Aunque todos los perros presentaron, al menos, una infección transmitida por garrapatas, en este estudio se evaluaron únicamente mascotas domiciliadas, que nunca estuvieron en situación de calle, y la mayoría de sus dueños tenía solo un animal en la vivienda (60 % de los participantes).

Los patrones de consumo y el estado de salud de los perros, también son factores de riesgo importantes para las enfermedades transmitidas por garrapatas. En diversos estudios, se ha señalado que el tipo de dieta predispone a los perros a infecciones por *E. canis*, en especial, a aquellos cuya alimentación se basa en restos de comida casera. Igualmente, se ha asociado el estado de salud del perro con el riesgo de infección por *Ehrlichia* spp. Los animales en deficientes condiciones de salud presentan una mayor probabilidad de desarrollar ehrlichiosis. Esta infección está estrechamente relacionada con el estado inmunológico canino que, a su vez, depende de aportes nutricionales que la comida casera no ofrece. Además, un estado de salud previo debilitado puede predisponer a infecciones de todo tipo, no solo a aquellas transmitidas por garrapatas ^52^^-^^54^.

La mitad de los perros estudiados se alimentaba de comida casera y la mayoría presentaba buenas condiciones de salud según sus dueños (70 %). Esta discrepancia respecto a lo reportado en otros estudios ^52^, se podría explicar por una composición adecuada de la dieta casera, capaz de cubrir las necesidades nutricionales de los animales. No obstante, la presencia de manifestaciones clínicas de enfermedades transmitidas por garrapatas - diagnosticadas en los perros-constituye un factor de riesgo para la infección por *Ehrlichia* spp. en los humanos ^52^.

Se demostró la presencia de garrapatas de cuerpo duro y blando en el 75,5 % (n = 37) de los perros infestados con al menos diez garrapatas en su superficie. Se considera que el número de garrapatas y el grado de infestación, son factores de riesgo para cualquier enfermedad transmitida por estos vectores. Esto podría explicar la diferencia encontrada por Yuasa *et al*., ^55^ quienes reportaron que *A. platys* es más prevalente en poblaciones caninas con altas tasas de infestación de *R. sanguineus*, hallazgo que fue confirmado también por Selim *et al.*^56^.

La mayoría de los perros estudiados dormía fuera del domicilio y el 56 % de sus dueños manifestó pasear a sus mascotas, factor que posiblemente favorece el contacto de los canes con áreas infestadas por garrapatas. Varios autores describen una mayor prevalencia de enfermedades transmitidas por garrapatas en cánidos con acceso libre a exteriores, como cazadores, perros en situación de calle e incluso aquellos que pasean, por su continua exposición al vector ^57^^-^^59^. Otros autores han propuesto que el contacto de humanos con perros previamente expuestos a zonas con cobertura gramínea es un factor de riesgo ^60^.

En este estudio, los sujetos que no paseaban a sus perros tenían mayor probabilidad de infectarse con *Babesia* spp. Esto podría explicarse por la permanencia prolongada del can en el peridomicilio, lo que favorecería la acumulación de vectores como *R. sanguineus* y, por consiguiente, un mayor grado de infestación ^45^.

El 90 % de la población canina estudiada no contaba con asistencia veterinaria, por consiguiente, solo el 10 % completó alguna terapia antibiótica contra estas enfermedades. Según Pérez-Macchi *et al*., ^53^ existe una asociación entre la ausencia de control veterinario y el riesgo de infección por *A. platys*. Este hallazgo destaca la necesidad de contar con asistencia veterinaria periódica, al menos una vez al año. Poco más de la mitad de los dueños de las mascotas manifestó aplicar métodos garrapaticidas a sus mascotas o en sus domicilios. Se ha reportado la ausencia de tratamiento garrapaticida como un factor de riesgo significativo para infecciones por *Anaplasma*^56^.

Se observó que visitar viviendas con perros está asociado con mayor probabilidad de infección por *Ehrlichia* spp. (p < 0,06). Esta relación puede deberse a una mayor exposición a la interfaz mascota-humano, ya que este contacto es clave para el desarrollo de infecciones por patógenos transmitidos por garrapatas. Estos resultados justifican la necesidad de realizar estudios más amplios en la zona que involucren especies de reservónos, hospederos y vectores.

A pesar de contar con una población de 105 personas (58 %) propietarias de perros, solo 10 de ellas (9,5 %) acudieron a la segunda convocatoria de esta investigación, lo que limitó la muestra del estudio. Por esta razón, se requiere ampliar el número de perros en la región para obtener conclusiones más representativas.

No se encontró una asociación entre el nivel socioeconómico y el riesgo de infecciones por patógenos transmitidos por garrapatas, aunque se ha demostrado que las condiciones socioeconómicas y culturales son un factor de riesgo para la infección por *A. phagocytophilum*^14^. Asimismo, se ha señalado que existen otros factores socioeconómicos que pueden aumentar la susceptibilidad de las poblaciones a desarrollar enfermedades transmitidas por vectores como la modernización del transporte comercial, las actividades económicas ilegales, los cambios en el uso de los suelos, la explotación acelerada de los recursos naturales y el deterioro de las infraestructuras públicas de salud ^61^.

Bayles y Alian reportaron que no hay una relación entre el grado de instrucción y una mayor incidencia de enfermedades transmitidas por garrapatas, hallazgo similar al del presente estudio. Sin embargo, evidenciaron que la pobreza actúa como potencial factor de riesgo en el contexto de la transmisión de estas infecciones ^62^.

En Chile, Acosta-Jammet *et al*. encontraron una asociación entre un bajo nivel de educación y la seropositividad para *Anaplasma*, lo que podría estar relacionado con un mayor grado de exposición derivado de condiciones socioeconómicas desfavorables ^63^. Tales resultados difieren de los obtenidos en el presente estudio, quizás por el número limitado de participantes categorizados en el estrato de pobreza o clase obrera (15 %).

La ciudad de Puerto Ordaz es una zona rica en diversidad de especies animales e incluso es escenario de uno de los más importantes movimientos migratorios de aves del planeta, ya que cada año en la Plaza de las Ciencias del Sur recibe alrededor de 500.000 golondrinas de río (*Progne tapera fusca*) y 3.000 atrapamoscas tijera (*Tyrannus savana*). Estas aves son capaces de liberar garrapatas infectadas al desplazarse y sirven como vehículos de amplia difusión de agentes bacterianos, parásitos y hongos. Además de las aves migratorias, otros animales observados en Ciudad Guayana y Ciudad Bolívar, como perros callejeros, gatos, zamuros, palomas, iguanas, roedores, entre otros, contaminan los suelos y las plantas con garrapatas ^47^^,^^64^, lo que representa un riesgo para la población humana.

Con respecto a las alteraciones observadas en los parámetros hematológicos, estas coinciden con las reportadas por otros investigadores ^3^, quienes señalan la leucopenia moderada y la trombocitopenia como hallazgos iniciales característicos en las infecciones por *Anaplasma* spp. y *Ehrlichia* spp. La anemia es un hallazgo común que generalmente ocurre después del descenso de los niveles de leucocitos y plaquetas. Cabe destacar que la leucopenia producida por las enfermedades transmitidas por garrapatas puede generar inmunosupresión a largo plazo, lo cual predispone al individuo a padecer múltiples enfermedades infecciosas que, junto con otras comorbilidades, pueden resultar fatales ^65^.

Los parámetros hematológicos en los casos de babesiosis varían según el grado de parasitemia. A menudo, esta infección se asocia con anemia hemolítica; el aumento de las enzimas hepáticas y los cambios en los recuentos leucocitarios y plaquetarios podrían servir de herramientas para el diagnóstico diferencial de otras enfermedades febriles ^22^. A pesar de ello, en esta investigación no se encontró asociación alguna entre los valores hematimétricos y las infecciones por *Ehrlichia* spp., *Anaplasma* spp. y *Babesia* spp., lo cual difiere de lo reportado en estudios previos.

Toda la población canina evaluada con examen físico (n = 10) presentó al menos una de las tres enfermedades transmitidas por garrapatas estudiadas. *Ehrlichia* fue el microorganismo más prevalente en todos los perros (n = 10) seguido de coinfecciones por *Anaplasma* (n = 5) y *Babesia* (n = 5). En contraste, otros investigadores han señalado predominio de la coinfección *Ehrlichia* spp. y *Anaplasma* spp. ^66^.

Algunos estudios han señalado la presencia solo de ehrlichiosis en perros enfermos mediante la técnica de reacción en cadena de la polimerasa (PCR) y la visualización directa de mórulas en linfocitos y monocitos ^67^. Mediante diferentes técnicas, también se ha demostrado en perros la presencia de múltiples patógenos transmitidos por vectores, como *Dirofilaria immitis, Ehrlichia* spp. y *Anaplasma* spp. ^68^. De manera similar a lo encontrado en el presente estudio, *Ehrlichia* spp. ha sido identificado como el principal patógeno transmitido por garrapatas en perros, seguido de *Anaplasma* spp. y *Babesia* spp.

Por su parte, otros estudios han reportado otras prevalencias en las que *Anaplasma* spp., se destaca como el principal patógeno en la población canina. En un estudio realizado en perros de la región del Caribe, en el que se emplearon técnicas de caracterización molecular, se identificó una prevalencia del 18,7 % de *A. platys* y del 16,8 % de *E. canis*^69^. En un estudio que incluyó 938 perros provenientes de todas las regiones de la República de Corea, se estimó una seroprevalencia del 15,1 % de *Anaplasma* spp., 10,3 % de *Ehrlichia* spp., 6,4 % de B. burgdorferi y 1,7 % de B. gibsonien ^70^. Lee *et al*. señalan que los perros coreanos tienen escasa exposición a *Babesia* spp., aunque otros autores han señalado a este patógeno como el principal agente causal de enfermedades transmitidas por garrapatas ^70^.

En la presente investigación, se reportó que la mayoría de los perros (80 %) tenía coinfecciones con los patógenos transmitidos por garrapatas. La más prevalente fue la triple coinfección por *Ehrlichia, Anaplasma y Babesia* (40 %), seguida de *Ehrlichia* y *Anaplasma* (20 %), y *Ehrlichia* y *Babesia* (20 %). El 20 % restante correspondió a perros infectados únicamente por *Ehrlichia* spp. Esta proporción difiere de la de otros estudios. Por ejemplo, Ramos *et al*. ^71^ describieron como coinfecciones más frecuentes, aquellas por *E. canis* y *A. platys* (16,09 %), seguidas de *E. canis* y B. canis (2,92 %), y *E. canis, A. platys* y *Babesia canis* (1,95 %) en perros del área metropolitana de Recife, Brasil. Estas frecuencias se deben probablemente a la capacidad de *R. sanguineus* de transmitir los tres patógenos, y a la ausencia de control veterinario y de antibioticoterapia en una población canina geriátrica.

En el presente estudio, se evidenció una importante prevalencia de infecciones transmitidas por garrapatas (85,1 %) en la población evaluada, principalmente por *Ehrlichia* spp. (85,1 %), *Babesia* spp. (29,8 %) y *Anaplasma* spp. (21,5 %). Estas infecciones son más probables en personas en contacto con perros geriátricos que frecuentan sitios al aire libre o infectados por patógenos transmitidos por garrapatas.
